# Would Repurposing Minocycline Alleviate Neurologic Manifestations of COVID-19?

**DOI:** 10.3389/fnins.2020.577780

**Published:** 2020-09-30

**Authors:** Aline C. Oliveira, Elaine M. Richards, Marianthi M. Karas, Carl J. Pepine, Mohan K. Raizada

**Affiliations:** ^1^Department of Physiology and Functional Genomics, College of Medicine, University of Florida, Gainesville, FL, United States; ^2^Division of Cardiovascular Medicine, Department of Medicine, College of Medicine, University of Florida, Gainesville, FL, United States

**Keywords:** neuroinflammation, SARS-CoV-2, minocycline, respiratory syndrome, autonomic system, microglia, COVID-19, hypoxia

## Introduction

Severe acute respiratory syndrome coronavirus-2 (SARS-CoV-2) is the etiologic agent of COVID-19 pandemic (Zhu et al., 2020). SARS-CoV-2 causes systemic infection varying in severity from asymptomatic, to mild (fever, cough, loss of smell and taste, leg pain, headache, diarrhea, fatigue), to multi-organ dysfunction/failure (Chen N. et al., 2020). Acute lung injury is the hallmark of COVID-19 and a subset of patients develop pneumonia and severe dyspnea requiring ICU admission. Most patients in critical condition have advanced age and pre-existing hypertension, cardiovascular disease (CVD), diabetes, obesity, and/or chronic lung conditions (Chen N. et al., 2020; Sharma et al., 2020b). Dyspnea and saturation of 90% or less despite oxygen supplementation is a major risk factor for fatal outcomes (Xie et al., 2020).

Angiotensin 1 converting enzyme 2 (ACE2) is the receptor that SARS-CoV-2 uses to enter host cells. ACE2 is highly expressed in lung but widespread distribution of ACE2 provides potential for infection of other organs. ACE2 is a member of the renin-angiotensin system (RAS) that plays an important role in the homeostasis of the cardiopulmonary system. The RAS consists of a vasoconstrictive, pro-inflammatory, and pro-fibrotic axis that includes angiotensin converting enzyme, angiotensin II and angiotensin type 1 receptor. Actions of this axis are balanced by the vasoprotective axis of the RAS that includes ACE2, angiotensin-(1-7) and MAS). Protective effects of ACE2 against cardiorespiratory diseases have been explored for 20 years (Sharma et al., 2020b).

After binding SARS-CoV-2, ACE2 is endocytosed downregulating cell surface ACE2. This explains the systemic RAS imbalance and increased pro-inflammatory Ang II observed in COVID-19 that intensifies multi-organ damage (Liu et al., 2020). Similarly, while ACE2-knock out mice develop severe acute respiratory distress, mice overexpressing ACE2 develop neither pulmonary hypertension (PH) nor neuroinflammation in a chronic hypoxia model. This illustrates the critical importance of ACE2 in balancing protective and pro-inflammatory effectors of RAS, by converting pro-inflammatory Ang II into beneficial Ang-(1-7). A randomized clinical trial evaluating efficacy, safety, and clinical impact of intravenous Ang-(1-7) vs. standard treatment in ICU patients with COVID-19 is underway (NCT04332666). Rationale for targeting the RAS to minimize systemic manifestations of COVID-19 are discussed in detail elsewhere (Sharma et al., 2020b).

## SARS-CoV-2 and the Central Nervous System

It is becoming increasingly evident that COVID-19 also affects the central nervous system (Asadi-Pooya and Simani, 2020; Chu et al., 2020; Kanberg et al., 2020; Li et al., 2020; Mao et al., 2020; Moriguchi et al., 2020; Reichard et al., 2020; Sellner et al., 2020; Solomon et al., 2020; Yashavantha Rao and Jayabaskaran, 2020). However, controversial results regarding direct or indirect effects of SARS-COV-2 in the brain highlight the complexity of this disease and may indicate unique responses in individuals with no discernible common pattern yet uncovered. *In vitro* studies show that SARS-CoV-2 not only infects, but also replicates in neurons, a phenomenon not observed during SARS-CoV infection (Chu et al., 2020). ACE2 is expressed throughout the CNS, in neurons, glial, endothelial, and arterial smooth muscle cells (Xia and Lazartigues, 2008), allowing virus to infect the brain and impair inter-and intra-cellular communication. An observational study of 214 COVID-19 patients reported 78 patients exhibiting three categories of neurologic manifestation: CNS effects such as dizziness, headache, impaired consciousness (somnolence, stupor, and coma), confusion and delirium in conscious patients, acute cerebrovascular disease, ataxia, seizures; peripheral nervous system effects such as taste, smell or vision impairments, nerve pain, and musculoskeletal injury (Mao et al., 2020). Among 64 consecutive hospitalized COVID-19 patients, 58 exhibited neurological symptoms (encephalopathy, agitation, confusion, and corticospinal tract signs) (Helms et al., 2020a). A recent MRI-based 3-month follow-up study demonstrates that 55% of patients who recovered from COVID-19 infection had neurological symptoms and exhibited significantly higher gray matter area in brain regions relevant to memory, smell, tremor etc. (Yiping et al., 2020). Together, these data indicate that neurological manifestations of COVID-19 are relatively frequent especially in patients with severe disease, raising questions about mechanism, chronic consequences, and management since neither a vaccine nor specific treatment is available. Evidence supports many routes for CNS effects of SARS-CoV-2. A common sign of SARS-CoV-2 infection is anosmia, resulting from infection of nasal epithelium and olfactory nerve damage. The virus may infect the CNS from this peripheral nerve by crossing synapses (Li et al., 2020) suggesting a peripheral to CNS route of viral transmission and damage. While one study showed that SARS-CoV-2 was not present in cerebrospinal fluid (CSF) of 7 patients with confirmed COVID-19 and neurological symptoms, another detected SARS-CoV-2 by RT-PCR in CSF of a 24 year-old man with meningitis/encephalitis (Moriguchi et al., 2020). This suggests viral entry from blood through the choroid plexus, but could also result by virus released from damaged cells in the brain into the CSF. More conclusively, post-mortem study of a 74 year-old man identified virus in the frontal lobe, with viral-like particles in neuronal cell bodies, small vesicles of endothelial cells, and throughout the cerebral microvasculature into neural tissue. This suggests a hematogenous route as a likely pathway to the brain (Paniz-Mondolfi et al., 2020). While evidence for the influence of COVID-19 in the brain continues to emerge, long-term consequences of this effect remains under investigation.

Nonetheless, plasma biomarkers of neuronal injury and glial activation are positively correlated with severity of COVID-19 symptoms (Kanberg et al., 2020) and were confirmed post-mortem (Reichard et al., 2020). Regardless of viral expression in brain, acute hypoxic ischemic damage was detected in brains of all 18 patients with SARS-CoV-2 infection (Solomon et al., 2020) and may indicate another route to induce neuroinflammation and increase inflammatory cytokines. This resembles the effects of hypoxia in animal models, which are microglia activation, neuroinflammation, autonomic imbalance and increased inflammatory cytokines leading, over time, to pulmonary hypertension (Savale et al., 2009; Oliveira et al., 2018; Sharma et al., 2020a).

In addition, patients with severe infection have increased inflammatory responses with CNS-related symptoms as the main form of neurologic injury, indicating indirect effects of SARS-CoV-2 infection in the brain, via pulmonary disease with increased pro-inflammatory cytokines (Mao et al., 2020). Comparing 113 deceased with 161 recovered patients revealed increased interleukin (IL) 2 receptor, IL-6, IL-8, IL-10, and tumor necrosis factor (TNF) and 20% with hypoxic encephalopathy amongst the deceased (Chen T. et al., 2020). Elevated IL-6 and abnormal permeability of blood meningeal barrier associated with encephalopathy was identified in another cohort with 140 patients admitted to ICU with COVID-19 diagnosis. Eighty-four percent showed abnormal neurological examination and/or delirium with significant worse prognosis compared to patients without delirium and normal neurological exam (Helms et al., 2020b). It might be explained by the intense systemic inflammatory response that leads to blood-brain barrier leakage and higher permeability to peripheral cytokines that exacerbate neuroinflammation and consequently, neurological damage (Cain et al., 2019). Bone marrow-derived cells may also contribute to neuroinflammation as demonstrated in hypertensive rat models. These cells migrate to the CNS and differentiate into microglia-like cells (Santisteban et al., 2015). Interestingly, bone marrow cells from ACE2 deficient mice promoted increases in inflammatory markers, which further contribute to neuroinflammation (Thatcher et al., 2012).

## Minocycline

Minocycline is part of the family of tetracycline antibiotics that possess both antibiotic and anti-inflammatory properties. Minocycline has been safely used for more than 40 years which produces beneficial effects in diverse pathophysiological conditions and is emerging as a useful drug in providing neuroprotection as a result of its ability of pass through the blood brain barrier (Garrido-Mesa et al., 2013). Although the precise mechanism of minocycline actions remain elusive, it appears that effects on multiple enzyme systems and pathways could account for its diverse actions. It inhibits bacterial protein synthesis by preventing aminoacyl-tRNA attachment to the ribosomal acceptor and downregulates the nuclear factor–κ B pathway. In addition, its inhibitory effects on key enzymes like inducible nitric oxide synthase, matrix metalloproteases, phospholipase A2, protein tyrosine nitration, caspases, and other apoptotic molecules, p38 map kinase, poly[ADP] ribose polymerase1 are linked with minocycline's anti-inflammatory, immunomodulatory, and neuroprotective actions (Garrido-Mesa et al., 2013). Minocycline is a highly lipophilic molecule; this property increases its half-life and allows excellent tissue penetration, and it easily crosses the BBB. Finally, minocycline inhibits HIV activation, proliferation and replication in microglia, macrophages, and lymphocytes (Garrido-Mesa et al., 2013).

**Figure 1:**
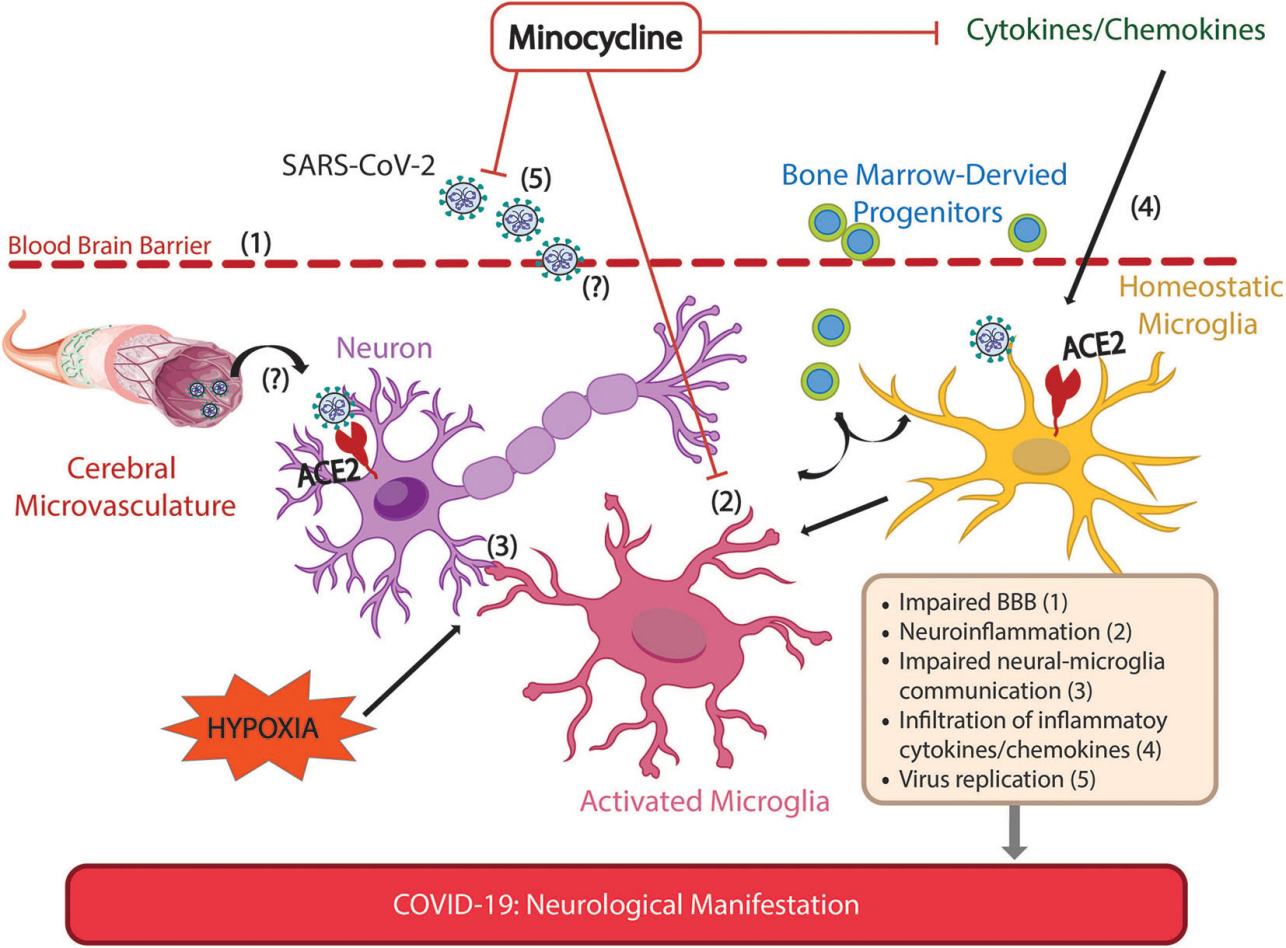
Possible sites of action of minocycline for alleviation of neurological manifestations of Covid-19. Neurological manifestations have been extensively reported in COVID-19 patients, and are associated with more severe symptoms. These manifestations may arise by direct and/or indirect mechanisms following SARS-CoV-2 infection. The SARS-CoV-2 receptor, ACE2, is expressed in neurons, glial and endothelial cells allowing the virus to infect and spread in the brain, impairing cellular communication. SARS-CoV-2 virus has been demonstrated in brain parenchyma and possible routes for SARS-CoV-2 infiltration such as impaired blood brain barrier are shown. Anosmia, a common sign of SARS-CoV-2 infection reflecting olfactory nerve damage, may illustrate another possible route of virus infiltration, via peripheral nerves to the CNS. Indirect effects on CNS include those from Covid-19-induced hypoxia such as neuroinflammation, impaired neuron-microglia communication, autonomic imbalance, impaired BBB, increased inflammatory cytokines and increased release of BM-derived progenitor cells. This is consistent with experiments in rodents that directly investigated brain damage due to hypoxia. Minocycline, an anti-inflammatory antibiotic that readily penetrates the CNS, counteracts neuroinflammation, virus replication and attenuates the increase of pro-inflammatory cytokines. Together, these actions of minocycline alleviate hypoxia-induced neuroinflammation and impaired neural-microglial communication that may precipitate neuronal and glial injury, thus preventing potential long-term neurological consequences of COVID-19.

We recently found neuroinflammation critical in driving PH and impaired brain-lung-gut communication responsible for PH-associated lung pathology; the number of activated microglia was positively correlated with PH severity (Oliveira et al., 2018; Sharma et al., 2018). This occurred in various PH models such as hypoxia, Sugen-hypoxia, and monocrotaline-induced PH in rats and mice. Moreover, we demonstrated that targeting neuroinflammation by inhibition of microglia activation could be a novel therapeutic strategy for PH. First, absence of CX3CR1 expression, a condition rendering microglia unable to activate, prevented neuroinflammation and PH induced by hypoxia (Oliveira et al., 2018). Secondly, minocycline, an anti-inflammatory antibiotic also used as a microglial inhibitor, significantly reduced the numbers and activation of microglia in the paraventricular nucleus of hypothalamus (PVN), reduced expression of cytokines such as IL-1β, IL-6, and TNF-α, attenuated lung inflammation, heart hypertrophy and sympathetic drive in monocrotaline-treated rats (Sharma et al., 2018). Significant numbers of severely ill COVID-19 patients have diabetes and hypertension, comorbidities where neuroinflammation is an important contributor. Minocycline demonstrated beneficial outcomes in diabetes-linked peripheral and autonomic neuropathy and cognitive impairment in rodents (Syngle et al., 2014; Ismail et al., 2019; Mehta and Banerjee, 2019). Our study with a small group of diabetic, obese and hypertensive patients, with minocycline used on a compassionate basis, showed consistent weight loss, improved HbA1c, improved neuropathic pain and impressive decrease in blood pressure (Yellowlees Douglas et al., 2012). Data from our ongoing trial (NCT02133885) indicate that minocycline decreases blood pressure, active microglia and plasma pro-inflammatory cells in treatment-resistant hypertensive patients. These observations led us to propose the following hypothesis (Figure 1): Minocycline attenuates microglia activation and arrests neuro-inflammation resulting in restoration of normal neuronal-microglia communication, controlling the pro-inflammatory profile especially in moderate and severe COVID-19 patients. This decreases the risk of developing long-term CNS consequences of SARS-CoV-2 infection. The following evidence supports our hypothesis: (i) Minocycline is a safe anti-inflammatory drug; (ii) In addition to anti-microbial activity, it is anti-inflammatory, antioxidant, inhibits ion channels and apoptosis and promotes neuronal regeneration in rodents (Miyachi et al., 1986; Rifkin et al., 1994; Tikka et al., 2001; Liu et al., 2007; Nutile-McMenemy et al., 2007). These actions, in addition to its ability to inhibit microglia are likely to contribute to beneficial effects in inflammatory diseases and CNS disorders including stroke (O'Dell, 1999; Naderi et al., 2020); (iii) minocycline has high lipid solubility, readily crossing the BBB; (iv) minocycline is the leading neuroprotective tetracycline, mainly by inhibition of microglia activation (Garrido-Mesa et al., 2013); (v) minocycline treatment effectively decreases inflammatory cytokines such as TNF, IL-1β, and IL-6 (Sharma et al., 2018), all highly expressed in COVID-patients and related to increased neurological damage (Chen N. et al., 2020); (vi) it is not an antiviral, however, it might act directly on the coronavirus by chelating zinc compounds on matrix metalloproteinase necessary for virus survival, infiltration and replication in the host (Sodhi and Etminan, 2020); (vii) alterations in fecal microbiota in Covid-19 patients were associated with severity and fecal levels of SARS-CoV-2 and gut dysbiosis persisted even after recovery (Zuo et al., 2020). These might also be attenuated by minocycline since it has been shown to modulate the composition of the gut microbiota and attenuate gut pathology in hypertension and depression (Schmidtner et al., 2019; Sharma et al., 2019; Yang et al., 2020); (viii) Its ability to reduce lung inflammation in PH and potentially beneficial effects in diabetes, obesity and hypertension are added bonuses. Finally, COVID-19 is emerging as a complex disease involving multiple organs and patients undergo multiple drug treatment to control diverse symptoms. Therefore, potential drug interactions should be considered before addition of minocycline since this anti-inflammatory antibiotic could produce adverse interactions with anti-viral drugs, other antibiotics and drugs acting on the central nervous system.

## Discussion

The rapid increase in COVID-19 has led to recognition that neurological manifestations are more frequent than initially suspected with unknown long-term nervous system consequences at this early stage of the pandemic. Hundreds of registered clinical trials are currently evaluating the extension of neurological manifestations in COVID-19 patients, with some of those aiming to long-term effects on brain functions (e.g., ClinicalTrials.gov Identifier: NCT04401449). It will be imperative to better understand whether SARS-CoV-2 infection increases the incidence of, or predisposes to, neurological and neurodegenerative disorders like cognitive impairment and dementia. We need to understand whether the risk applies only to patients with neurological symptoms during initial infection or to all infected patients. This is of concern because while only 11% of rats infected with a related coronavirus exhibited neurological symptoms, 40% of animals without clinically recognized neurological signs had pathological brain lesions 8-months after infection (Nagashima et al., 1979). This suggests that effects of SARS-CoV-2 infection on the CNS require very close attention, particularly because long-term CNS-effects may emerge in patients without neurological symptoms during initial infection. Therapies that could minimize these consequences would be very valuable.

In conclusion, we believe that not only is there is an urgent need for effective therapies to minimize systemic effects of SARS-CoV-2 and its pro-inflammatory profile, but also to mitigate its CNS effects that are potentially long-term. Minocycline has effective mechanisms to protect the CNS, it attenuates neuroinflammation, counteracts the cytokine storm, modulates gut microbiome, and might inhibit viral replication. If confirmed, our hypothesis may represent an important contribution to prevent neurological impacts of infection and improve COVID-19 management.

## Author Contributions

AO contributed substantially to the conception, design, and writing the opinion letter. ER contributed to the writing and provided critical revision. MK contributed to the final figure. CP revised it critically for important intellectual content. MR contributed the draft and revised it critically for important intellectual content. All authors read and approved the final manuscript.

## Conflict of Interest

The authors declare that the research was conducted in the absence of any commercial or financial relationships that could be construed as a potential conflict of interest.
